# Current update on the protective effect of epicatechin in neurodegenerative diseases

**DOI:** 10.17179/excli2022-5034

**Published:** 2022-06-24

**Authors:** Riya Thapa, Gaurav Gupta, Piyush Dave, Santosh Kumar Singh, Abhay Raizaday, Waleed Hassan Almalki, Govind Vyas, Sachin Kumar Singh, Kamal Dua, Yogendra Singh

**Affiliations:** 1 School of Pharmacy, Suresh Gyan Vihar University, Mahal Road, Jagatpura 302017, Jaipur, India; 2 Department of Pharmacology, Saveetha Dental College, Saveetha Institute of Medical and Technical Sciences, Saveetha University, Chennai, India; 3 Uttaranchal Institute of Pharmaceutical Sciences, Uttaranchal University, Dehradun, India; 4 Department of Pharmacology, College of Pharmacy, Umm Al-Qura University, Makkah, Saudi Arabia; 5 Inva-Health Inc, Cranbury, NJ 08512, USA; 6 School of Pharmaceutical Sciences, Lovely Professional University, Phagwara, Punjab, 144411, India; 7 Faculty of Health, Australian Research Centre in Complementary and Integrative Medicine, University of Technology Sydney, Ultimo, NSW 2007, Australia; 8 Discipline of Pharmacy, Graduate School of Health, University of Technology Sydney, NSW 2007, Australia; 9 Department of Pharmacology, Maharishi Arvind College of Pharmacy, Ambabari Circle, Ambabari, Jaipur, 302023, India

## ⁯

**Figure 1:**
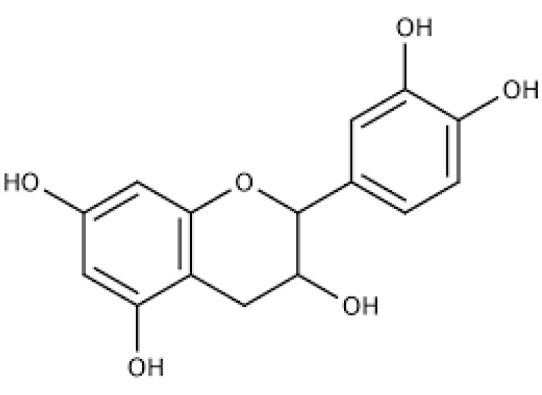
Chemical structure of epicatechin

Neurodegenerative diseases are characterized by the progressive loss of neural structures instead of the selective neuronal loss caused by metabolic or toxic disorders. Alzheimer's, Parkinson's, Huntington's, and amyotrophic lateral sclerosis are among the several neurodegenerative diseases for which there is no treatment (Ruz et al., 2020[21]). New and better treatment strategies are urgently required to tackle these fatal illnesses. For example, epicatechin is one of the most prevalent and plentiful flavonoids (Figure 1(Fig. 1)). Numerous organs and tissues, including the heart, skeletal muscle, and neurons, have been studied, and epicatechin has been associated with mitochondrial improvement (Panneerselvam et al., 2013[19]). Epicatechin has been demonstrated to aid in treating neurodegenerative diseases, although there is little data to back this claim (Shaki et al., 2017[22]). The discoveries will also offer researchers a roadmap for developing neuroprotective drugs that are safe and effective (Table 1(Tab. 1); References in Table 1: Al-Amri et al., 2013[1]; Ali et al., 2021[2], 2022[3]; Avramovich-Tirosh et al., 2007[4]; Beasley et al., 2019[5]; Bitu Pinto et al., 2015[6]; Cano et al., 2021[7]; Cuevas et al., 2009[8]; Diaz et al., 2019[9]; Ehrnhoefer et al., 2006[10]; Ferruzzi et al., 2009[11]; Koh et al., 2006[12]; Kumar and Kumar, 2009[13]; Li et al., 2004[14]; Lim et al., 2013[15]; Mandel et al., 2004[16]; Nan et al., 2021[17]; N'Go et al., 2021[18]; Rubio-Osornio et al., 2015[20]; Shaki et al., 2017[22]; Siddique et al., 2014[23]; Tseng et al., 2020[24]; Wang et al., 2012[25]; Xu et al., 2006[26]; Ye et al., 2012[27]; Zhou et al., 2019[28]).

**Figure 1:** Chemical structure of epicatechin

| Neurodegenerative diseases | Key findings | References |
|---|---|---|
| **Alzheimer Disease (AD)** | Cuevas et al. investigated Epicatechin's (EC) antioxidant effects on Aβ 25-35-induced brain damage *in vivo*. AD neurodegeneration is linked to Amyloid-beta (Aβ). Aβ 25-35 led to a considerable rise in lipid peroxidation (LPO) and reactive oxygen species (ROS) and reduced memory functions. Furthermore, results showed EC treatment prevented oxidative damage to the hippocampus induced by Aβ 25-35. | Cuevas et al., 2009 |
| | Ali et al. looked at the role of EC, Vitamin E, Vitamin C, and Se in improving the potential impact of physical and mental activities (Ph&M) over socially isolated and protein malnourished (SI&PM) as risk factors for Alzheimer's disease development in rats. In the AD, SI&PM, and SI&PM/AD groups, the combination of EC, VE, VC, and Se with Ph&M boosted brain monoamines, SOD, TAC, and BDNF. In addition, SI&PM-induced AD risk was reduced when antioxidants were combined with Ph&M activities. | Ali et al., 2021 |
| | The impact of EC on the memory function of AD rats was studied by Nan et al. After the AD rats were given EC, they spent more time in the target quadrant, demonstrating that EC may reduce Tau hyperphosphorylation, downregulate BACE1 and Aβ1-42 expression, and boost AD rats' antioxidant system as well as their cognition and memory. | Nan et al., 2021 |
| | Using isolated rat hippocampus mitochondria *in vivo*, Shaki et al. examined the effect of EC on mitochondrial damage produced by homocysteine (Hcy). EC decreased LPO and ROS levels while raising GSH levels concurrently. It has been shown that EC protects against oxidative stress, reduces mitochondrial damage, and cures neurological diseases caused by Hcy, including Alzheimer's disease. | Shaki et al., 2017 |
| | Diaz and colleagues investigated the effects of EC on Aβ25-35 neurotoxicity on spatial memory and the interaction between HSP immunoreactivity in the CA1 area of the rat HP. Treatment with EC reduces the risk of Alzheimer's disease. EC improves spatial memory performance by reducing A25-35-induced neurotoxicity, HSP-60, -70, and -90 immunoreactivity, and neuronal loss in the CA1 region of the Hp of A25-35-injected rats. | Diaz et al., 2019 |

EXCLI Journal 2022;21:897-903 – ISSN 1611-2156
Received: May 12, 2022, accepted: June 14, 2022, published: June 24, 2022

| | | |
|---|---|---|
| | Wang et al. studied that 3′-O-methyl-epicatechin-5-O-glucuronide was discovered for the first time in a biosynthetic EC metabolite and that proanthocyanidin (PAC) metabolites found in the brain monomeric (Mo) therapy increase baseline synaptic transmission in hippocampal slices via mechanisms associated with CREB signaling. | Wang et al., 2012 |
| | N'Go et al. investigated whether natural components from Chrysophyllum perpulchrum, such as EC and two dimeric procyanidins (EC + hexose), potentially inhibit the development of oxidative stress and cognitive abnormalities in a rat model of AD generated by Aβ1-40 injection into the CA1 region of the hippocampi. A rat's identification memory and spatial learning were much weaker. This was linked to an increase in Iba 1 immunoreactivity and NO levels in microglia. In the hippocampus, prefrontal cortex, and septum of AD-like animal models, malondialdehyde and SOD levels were associated, but not thiol content. | N'Go et al., 2021 |
| | Ferruzzi et al. found that frequent exposure to Grape seed proanthocyanidin extract (GSPE) enhanced bioavailability. In the brain tissues of rats given a single dose of GSPE, neither EC nor catechin (C) was found. Repeated delivery of GSPE seems to affect the accumulation of GA, C, and EC in the brain. | Ferruzzi et al., 2009 |
| | Vinpocetine, alone or in combination with EC, CoQ10, or VE & Se, was investigated for its possible neuroprotective effect and mechanism of action in reducing aluminum chloride-induced AD in rats by Ali et al. Histopathological examinations and DNA fragmentation tests revealed that the combination of Vinpocetine and EC exhibited the most incredible neuroprotective effects, protecting rat neurons against AD induced by AlCl3. | Ali et al., 2022 |
| | The research of Lim et al.aimed to find out if treating transgenic (Tg) mice with EC, a radical scavenger, improved AD symptoms. GTC improves AD characteristics, suggesting that it might be used to treat AD. | Lim et al., 2013 |
| **Parkinson's Disease (PD)** | Bitu Pinto et al. examined the neuroprotective properties of EC in a rat with PD. Results indicated that the neuroprotective effects of EC are most likely attributable to their considerable antioxidant and anti-inflammatory properties, emphasizing their potential for PD prevention and therapy. | Bitu Pinto et al., 2015 |
| | Tseng et al. investigated the protective benefits of EC against ROT-induced motor and neurochemical dysfunctions in rats. EC treatment decreased ROT-induced NO levels and LPO production; increased the activity of succinate dehydrogenase (SDH), ATPase, ETC enzymes as well as catecholamine levels in the striatum; and decreased neuroinflammatory and apoptotic levels, indicating that EC may play a clinically significant role in delaying or treating human PD. | Tseng et al., 2020 |
| | Zhou et al. investigated the protective impact of EC on apoptosis and the mTOR/AKT/GSK-3 pathway in substantia nigra neurons in 6-dopamine-induced PD ratsl. In rats with PD caused by 6-OHDA, the findings indicated that EC might inhibit neuronal cell death in the substantia nigra. | Zhou et al., 2019 |
| | Rubio-Osornio et al. discovered no hepatotoxicity in adult Sprague–Dawley rats with 50 mg/kg EC. According to this investigation, EC possesses neuroprotective properties in the MPTP-PD mouse model. EC might provide neuroprotection against Parkinson's disease. | Rubio-Osornio et al., 2015 |

EXCLI Journal 2022;21:897-903 – ISSN 1611-2156
Received: May 12, 2022, accepted: June 14, 2022, published: June 24, 2022

| | | |
|---|---|---|
| | Ye et al. evaluated highly differentiated PC12 cells treated with MPP(+) as an *in vitro* cell model to assess cell survival after EC treatment. SIRT1/PGC-1 is one of the pathways by which EC inhibits MPP(+)-induced cell damage in PC12 cells. | Ye et al., 2012 |
| | Al-Amri and co-authors aimed to determine if EC might inhibit the production of inflammatory mediators and protect dopaminergic neurons from LPS-induced neurotoxicity. Antioxidant EC was shown to have a possible therapeutic effect against LPS-induced neurotoxicity by decreasing TNF-alpha and NO inflammatory mediators in the midbrain while preserving DA levels. | Al-Amri et al., 2013 |
| | According to a study by Li et al., both the human dopaminergic cell line SH-SY5Y and primary rat mesencephalic cultures were significantly protected against microglial activation-induced neuronal injury by EC. The results indicate that EC is a potent inhibitor of microglial activation, suggesting that it might be employed to treat microglia-mediated dopaminergic neuronal damage in Parkinson's disease. | Li et al., 2004 |
| | The impact of EC on climbing ability, LPO, and apoptosis in the brains of PD model flies was explored by Siddique et al. The administration of 0.25, 0.50, and 1.0 g/mL of EC to the brains of PD model flies in a dose-dependent way; it reduced oxidative stress and apoptosis while preventing the loss of climbing ability. | Siddique et al., 2014 |
| **Huntington's Disease** | Kumar and Kumar (2009) demonstrated the effects of lycopene and EC on memory impairment and how 3-NP therapy disrupts the glutathione system. Treatments with lycopene and EC restored glutathione system function and dramatically enhanced memory. | Kumar and Kumar, 2009 |
| | The green tea polyphenol EC prevents mutant htt exon 1 protein from aggregating in a dose-dependent way. *In vitro*, EC contains mutant htt exon 1 protein from misfolding and oligomerizing, indicating that it interferes with early aggregation processes. According to their findings, EC, a modulator of htt exon 1 misfolding and oligomerization, may be able to attenuate polyQ-mediated toxicity *in vivo*. | Ehrnhoefer et al., 2006 |
| | The concept, that the presence of lipid vesicles affected the function of EC, was examined by Beasley et al. Curcumin was prevented from suppressing the formation of htt fibrils by adding 1-palmitoyl-2-oleoyl-glycerol-3-phosphocholine or vesicles generated from a whole-brain lipid extract. These findings suggest that EC and other htt exon 1 misfolding and oligomerization modulators might lower polyQ-mediated toxicity *in vivo*. | Beasley et al., 2019 |
| | Cano et al. claim that ascorbic acid was used to integrate EC into PEGylated poly(lactic-co-glycolic acid) NPs. Intoxication with 3-nitropropionic acid caused HD-like striatal lesions and motor deficits in mice. Motor abnormalities and depressive-like behavior related to 3-nitropropionic acid poisoning were considerably reduced by EC/AA NPs than by free EC. Treatment with EC/AA NPs also reduced neuroinflammation and stopped neuronal loss. | Cano et al., 2021 |
| | According to AvramovichTirosh et al., M-30 and EC reduced apoptosis in human SH-SY5Y neuroblastoma cells in a neurorescue, serum deprivation model via multiple protection mechanisms. These mechanisms included the reduction of pro-apoptotic proteins and the promotion of | Avramovich-Tirosh et al., 2007 |

| | | |
|---|---|---|
| | morphological changes. In addition, these changes resulted in axonal growth-associated protein-43 (GAP-43), which was implicated in neuronal differentiation. | |
| **Lewy Body Disease** | Iron is essential for the pathophysiology of oxidative stress, which involves the death of dopaminergic neurons and the degradation of proteins via ubiquitination, highlighting the relevance of iron in these processes. In rats and non-human primates, iron and -synuclein accumulation in the SNpc is linked to MPTP-induced neurodegeneration. In MPTP-induced dementia, the iron buildup has been connected to the ubiquitination of iron regulatory proteins, related to NO-dependent mechanisms. The buildup of iron and -synuclein in the SNpc of mice and rats is inhibited by EC and other radical scavengers. These radical scavengers protect the nervous system against neurotoxins. | Mandel et al., 2004 |
| **Amyotrophic Lateral Sclerosis (ALS)** | In a transgenic mouse model of ALS, Xu and colleagues investigated the neuroprotective effects of EC. SOD1-G93A transgenic mice and wild-type mice were separated into EC-treated and vehicle-treated control groups at random intervals. Oral EC treatment started at a pre-symptomatic stage in a mouse model of ALS dramatically delayed illness onset and increased life duration. This research adds to the expanding amount of data that EC has various medicinal properties. | Xu et al., 2006 |
| | According to Koh and colleagues, the impact of EC on ALS model mice with the human G93A mutant Cu/Zn-SOD1 gene, more than 2.9 micrograms of EC per gram of body weight prolonged symptom onset and duration of life, preserved more survival signals, and reduced death signals. These findings suggest that EC might be a disease-modifying treatment for persons with ALS. | Koh et al., 2006 |

## *Conflict of interest*

The authors declare no conflict of interest.

## Conflict of interest

The authors declare no conflict of interest.

## Figures and Tables

**Table 1:**
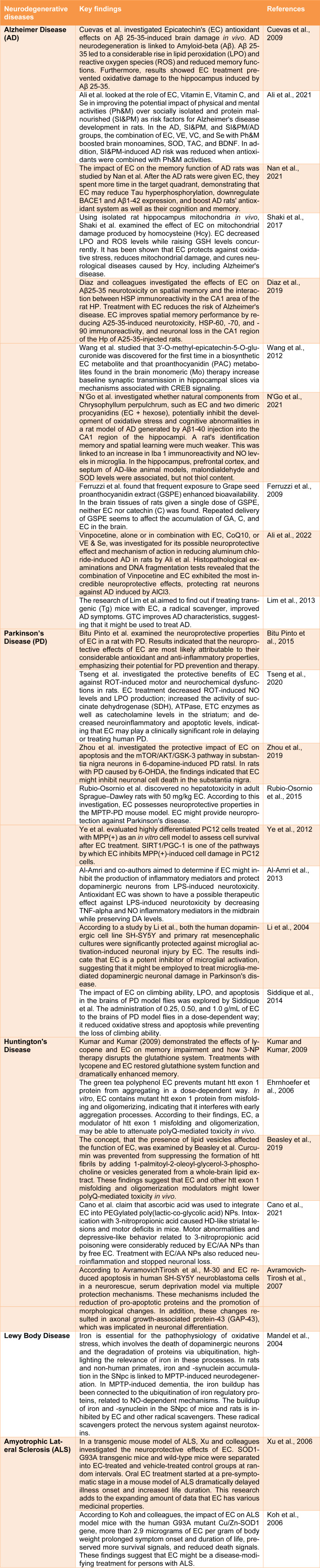
An update on the protective effect of epicatechin in various neurodegenerative diseases
